# Yield effect of applying earthworm castings produced during the oilseed rape-growing season in rice-oilseed rape cropping fields to rice

**DOI:** 10.1038/s41598-018-29125-y

**Published:** 2018-07-17

**Authors:** Min Huang, Chunrong Zhao, Yingbin Zou, Norman Uphoff

**Affiliations:** 1 Southern Regional Collaborative Innovation Center for Grain and Oil Crops (CICGO), Hunan Agricultural University, Changsha, 410128 China; 2 International Programs-College of Agriculture and Life Sciences (IP-CALS), Cornell University, Ithaca, 14853 USA

## Abstract

In-field earthworm density can be increased by planting oilseed rape during the non-rice growing season as compared to maintaining the rice-growing fields in fallow. This study was conducted to determine the effect on rice yield of earthworm castings produced during the oilseed rape-growing season in rice-oilseed rape cropping fields and to identify the critical factors that contribute to the yield effect. Field microplot experiments were conducted in 2016 and 2017. In 2016, a rice cultivar was grown under a factorial combination of absence (EC_0_: 0 kg m^−2^) and presence of earthworm castings (EC_1_: 17 kg m^−2^) with three N application rates (9, 12 and 15 g m^−2^). In 2017, nine rice cultivars were grown under EC_0_ and EC_1_ with the moderate N rate as was used in 2016. Results showed that application of earthworm castings produced during the oilseed rape-growing season in rice-oilseed rape cropping fields had a significant positive yield effect on rice. This was attributed to increased panicle m^−2^ and total aboveground biomass while spikelets panicle^−1^, spikelet filling percentage, grain weight, and harvest index were not affected. Our study indirectly provides a new evidence that oilseed rape is an excellent previous crop for cereals.

## Introduction

Rice is a major staple food for almost 50% of the world’s population, and rice fields account for more than 12% of the global cropland area^1^. Of these rice fields, nearly 90% are located in Asia (World Rice Statistics database, available at http://ricestat.irri.org:8080/wrs, hereinafter referred to as WRS database). In order to produce enough food for rapidly growing populations, agricultural land use in Asia has become very intense^2^. In the past 50 years, the intensification of rice-based cropping systems has helped ensure production of sufficient rice and other food crops^3^. However, the continuous intensive practice of rice-based cropping systems such as rice-wheat cropping system practiced for several decades has led to declines in productivity and raised concerns about sustainability^4^.

China is one of the major rice-producing countries in the world with an area of about 30 million hectares, accounting for approximately 20% of the world’s rice field area (WRS database). Rice-based cropping systems are diverse in China due to differences in agro-climatic zones^5^. Among those cropping systems, rice-wheat and rice-oilseed rape are two long-established major ones^6^. In contrast to the concerns about sustainability of continuous rice-wheat cropping system as mentioned above, our study suggests that long-term rice-oilseed rape cropping system can increase soil fertility and consequently reduce the dependence on external nitrogen (N) inputs and adverse impacts on the environment^7^. This supports the viewpoint that oilseed rape is an excellent and sustainable previous crop for cereals^8–10^.

**Figure 1:**
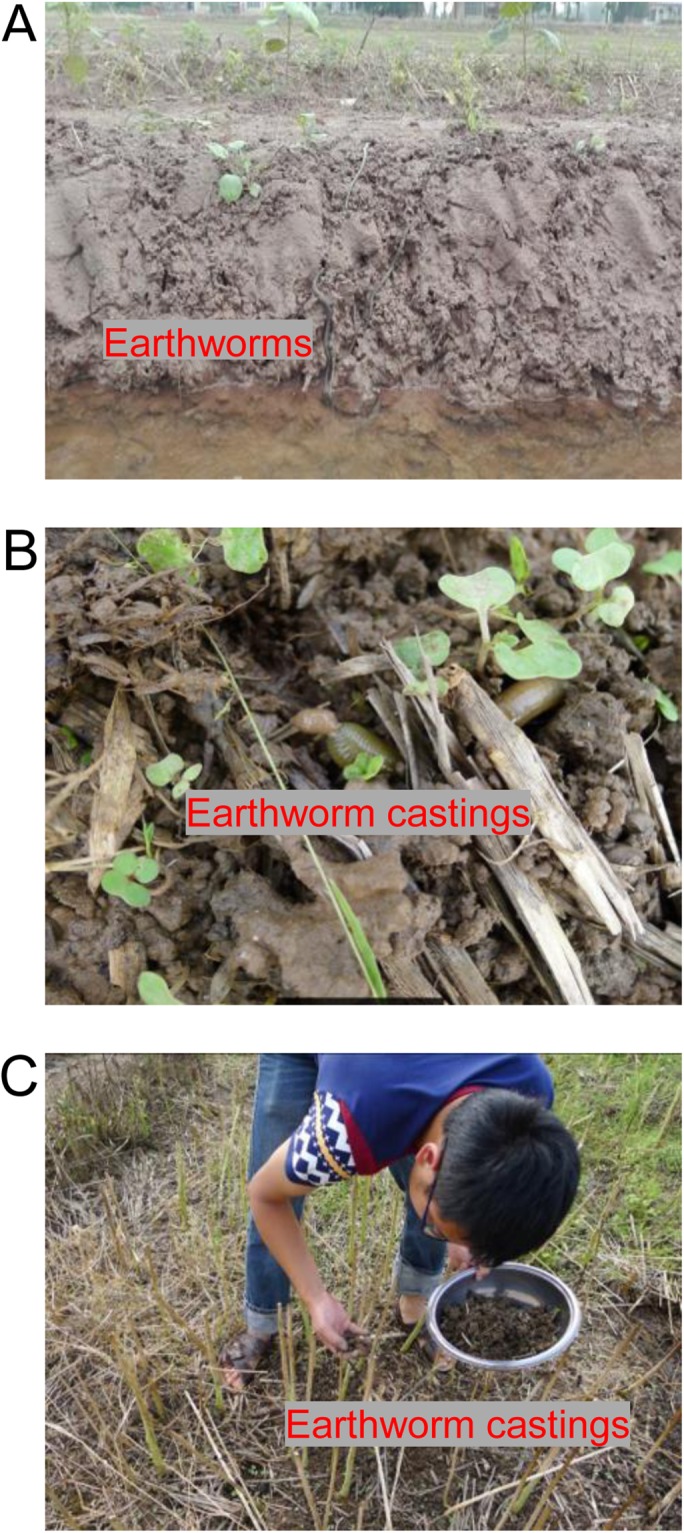
Earthworms migrated away from the rice field after flooding (**A**), earthworm castings produced in the oilseed rape-growing season (**B**), and earthworm castings collected after harvesting the oilseed rape (**C**). These photos were taken from rice-oilseed rape cropping fields located in Nanxian, Hunan Province, China in 2016.

Such a viewpoint also can be supported by our recent investigations which showed that in-field earthworm density during the non-rice growing season was doubled by planting oilseed rape as compared to fallow in long-term, no-tillage rice-based fields^11^. Although the earthworms are likely to migrate away from fields during the early stage of rice-growing when rice fields are flooded (Fig. 1A), their castings will remain in the fields (Fig. 1B). These facts indicate that growing oilseed rape as a previous crop for rice can enhance earthworm activity and hence may provide similar benefits as the option of applying the earthworm castings as fertilizer, especially in regions that are kept on fallow during the non-rice growing season. This aroused our interest to investigate the effect of the earthworm castings produced during the oilseed rape-growing season in rice-oilseed rape cropping fields on grain yield in rice. As a consequence, we conducted a pot experiment and found that grain yield was increased in a rice cultivar with application of the earthworm castings collected from oilseed rape fields^12^. However, this finding could be subject to the constraints of pot cultivation and cultivar specificity. Therefore, more experimentation should be done under field conditions with various rice cultivars to obtain more concrete results.

Rice yield is determined by sink size (spikelets per unit land area), spikelet filling percentage, and grain weight. Sink size is considered as the primary determinant of the rice yield, and it can be increased either by increasing the number of panicles or panicle size (spikelets per panicle), or both^13^. Because a strong compensation mechanism exists between the two yield components, a concurrent increase in them is not easy to achieve^14,15^. Increasing panicle size is a common approach for the rice breeders to enhance the sink size and consequently to improve rice yield potential^16^. However, there has contradictory statement that more panicle number should be emphasized to achieve higher grain yield in the super hybrid rice with large panicle size^17^.

In another approach, grain yield of rice is a function of total aboveground biomass and harvest index. It is generally accepted that achieving greater rice yields depends on increasing total aboveground biomass, because there is little scope to achieve further increases in the harvest index under favorable conditions^18–20^. The harvest index of modern high-yielding rice is around 50%^21^. However, in recent years there have been reports that high grain yield can achieved in rice with high harvest index^22,23^.

In our present study, grain yield and yield attributes in rice were compared between with and without applications of earthworm castings that were produced during the oilseed rape-growing season in rice-oilseed rape cropping fields in two-year field microplot experiments. Our objectives were to (1) determine the effect on rice yield of earthworm castings produced during the oilseed rape-growing season in rice-oilseed rape cropping fields, and (2) identify the critical factors that contributed to this yield effect.

## Results

In 2016, grain yield was significantly affected by earthworm casting treatment, but not by N rate and the interaction between earthworm casting treatment and N rate (Table 1). In 2017, earthworm casting treatment and cultivar had a significant effect on grain yield, while the interaction effect on grain yield between earthworm casting treatment and cultivar was not significant. Therefore, only the means of earthworm casting treatments in 2016 and the means of earthworm casting treatments and cultivars in 2017 were presented in subsequent tables for evaluating effect on yield attributes, making interpretation easier.

**Table 1:** Grain yields (g m^−2^) from a rice cultivar Liangyoupeijiu grown under absence (EC_0_: 0 kg m^−2^) and presence of earthworm castings (EC_1_: 17 kg m^−2^) with three N rates (N_1_: 9 g m^−2^; N_2_: 12 g m^−2^; and N_3_: 15 g m^−2^) in 2016, and from nine rice cultivars grown under EC_0_ and EC_1_ with one N rate (N_2_) in 2017.

N rate/cultivar	Earthworm casting treatment		
	EC_0_	EC_1_	Mean^†^
2016			
N_1_	846 (110)	1165 (116)	1006
N_2_	884 (18)	1178 (73)	1031
N_3_	932 (64)	1160 (41)	1046
Mean	887	1168	
Analysis of variance			
Earthworm casting treatment (EC)	**		
N rate (N)	ns		
EC × N	ns		
2017			
Guihefeng	752 (63)	1002 (55)	877c
Huanghuazhan	933 (54)	1113 (82)	1023b
Liangyoupeijiu	967 (78)	1176 (102)	1072b
Longliangyou 97	1157 (59)	1289 (84)	1223a
Shenliangyou 5814	1028 (28)	1315 (43)	1172a
Xiangliangyou 396	1084 (43)	1306 (97)	1195a
Y-liangyou 1	1035 (39)	1323 (74)	1179a
Y-liangyou 2	1142 (119)	1293 (21)	1218a
Zhunliangyou 608	1159 (42)	1273 (61)	1216a
Mean	1029	1232	
Analysis of variance			
EC	**		
Cultivar (C)	**		
EC × C	ns		

Values in parenthesis are the standard deviations.

^†^Means of cultivars with the same letters are not significantly different at the 0.05 probability level (LSD test).

** represents significance at the 0.01 probability level.

ns denotes non-significance at the 0.05 probability level.

EC_1_ had, respectively, 32% and 20% higher grain yield than EC_0_ in 2016 and 2017 (Table 1). In 2017, Longliangyou 97 produced the highest grain yield, although this was not significantly higher than from Shenliangyou 5814, Xiangliangyou 396, Y-liangyou 1, Y-liangyou 2 and Zhunliangyou 608, but it was 14–20% higher than the yields from Liangyoupeijiu and Huanghuazhan, and 39% higher than that from Guihefeng.

Panicles m^−2^ under EC_1_ were 21% and 13% higher than those under EC_0_ in 2016 and 2017, respectively (Table 2). There was no significant difference in spikelets panicle^−1^ between EC_1_ and EC_0_ in either 2016 and 2017. EC_1_ had higher spikelets m^−2^ than EC_0,_ by 27% in 2016 and by 16% in 2017. The differences in spikelet filling percentage and in grain weight were insignificant between EC_1_ and EC_0_ in both years.

**Table 2:** Yield components in rice as affected by earthworm castings in 2016, and by earthworm castings and cultivars in 2017.

Earthworm casting treatment/cultivar^†^	Panicles m^−2^	Spikelets panicle^−1^	Spikelets m^−2^ (×10^3^)	Spikelet filling (%)	Grain weight (mg)
2016^‡^					
EC_0_	192b	251a	48.2b	70.1a	22.8a
EC_1_	233a	262a	61.0a	71.4a	23.1a
2017					
EC_0_	247b	219a	53.1b	79.8a	21.3a
EC_1_	280a	222a	61.5a	80.7a	21.8a
Guihefeng	198e	293a	57.8ab	79.7bc	16.6e
Huanghuazhan	296ab	203c	60.2ab	82.2b	17.9e
Liangyoupeijiu	269bc	208c	55.9b	71.2d	23.4b
Longliangyou 97	298a	207c	61.6ab	77.2c	22.7bc
Shenliangyou 5814	263cd	212c	55.3bc	87.7a	21.0cd
Xiangliangyou 396	292ab	201c	58.8ab	79.2bc	22.6bc
Y-liangyou 1	280abc	195c	54.6bc	82.4b	22.5bc
Y-liangyou 2	239d	267b	63.8a	79.8bc	20.7d
Zhunliangyou 608	239d	200c	47.6c	83.1b	26.5a

Within a column for earthworm casting treatments in each year and for cultivars in 2017, data with the same letters are not significantly different at the 0.05 probability level (LSD test).

^†^EC_0_ and EC_1_ represent absence (0 kg m^−2^) and presence of earthworm castings (17 kg m^−2^), respectively.

^‡^Data are the means across three N rates in 2016.

In 2017, Longliangyou 97 had the highest panicles m^−2^, and similar values were observed in Huanghuazhan, Xiangliangyou 396 and Y-liangyou 1 (Table 2). The lowest panicles m^−2^ was recorded in Guihefeng. This variety also had the highest spikelets panicle^−1^, followed by Y-liangyou 2 and the other cultivars, which were not significantly different from one another. Y-liangyou 2 had the highest spikelets m^−2^, but this was not significantly different from the spikelets m^−2^ for Guihefeng, Huanghuazhan, Longliangyou 97 and Xiangliangyou 396, whereas Zhunliangyou 608 had the lowest spikelets m^−2^. Spikelet filling percentage was highest in Shenliangyou 5814 and lowest in Liangyoupeijiu. Zhunliangyou 608 had the highest grain weight, while Guihefeng and Huanghuazhan had the lowest grain weight.

EC_1_ produced 27% higher total aboveground biomass than EC_0_ in 2016 and 19% more in 2017 (Table 3). There was no significant in harvest index between EC_1_ and EC_0_ in either year. In 2017, Y-liangyou 2 produced the highest total aboveground biomass, but this was not significantly different from the biomass in Longliangyou 97, Shenliangyou 5814, Y-liangyou 1, and Zhunliangyou 608, while Guihefeng had the lowest total aboveground biomass. Xiangliangyou 396 had the highest harvest index, but similar values were recorded in Huanghuazhan, Longliangyou 97, Y-liangyou 1 and Zhunliangyou 608. The lowest harvest index was observed in Guihefeng.

**Table 3:** Total aboveground biomass and harvest index in rice as affected by earthworm castings in 2016, and by earthworm castings and cultivars in 2017.

Earthworm casting treatment/cultivar^†^	Total aboveground biomass (g m^−2^)	Harvest index (%)
2016^‡^		
EC_0_	1669b	45.9a
EC_1_	2097a	47.9a
2017		
EC_0_	1656b	53.4a
EC_1_	1970a	53.7a
Guihefeng	1478e	51.1e
Huanghuazhan	1641d	53.6abc
Liangyoupeijiu	1799c	51.3de
Longliangyou 97	1897ab	55.6ab
Shenliangyou 5814	1889abc	53.4bcd
Xiangliangyou 396	1844bc	55.7a
Y-liangyou 1	1879abc	53.9abc
Y-liangyou 2	1972a	53.0cde
Zhunliangyou 608	1920ab	54.5abc

Within a column for earthworm casting treatments in each year and for cultivars in 2017, data with the same letters are not significantly different at the 0.05 probability level (LSD test).

^†^EC_0_ and EC_1_ represent absence (0 kg m^−2^) and presence of earthworm castings (17 kg m^−2^), respectively.

^‡^Data are the means across three N rates in 2016.

**Figure 2:**
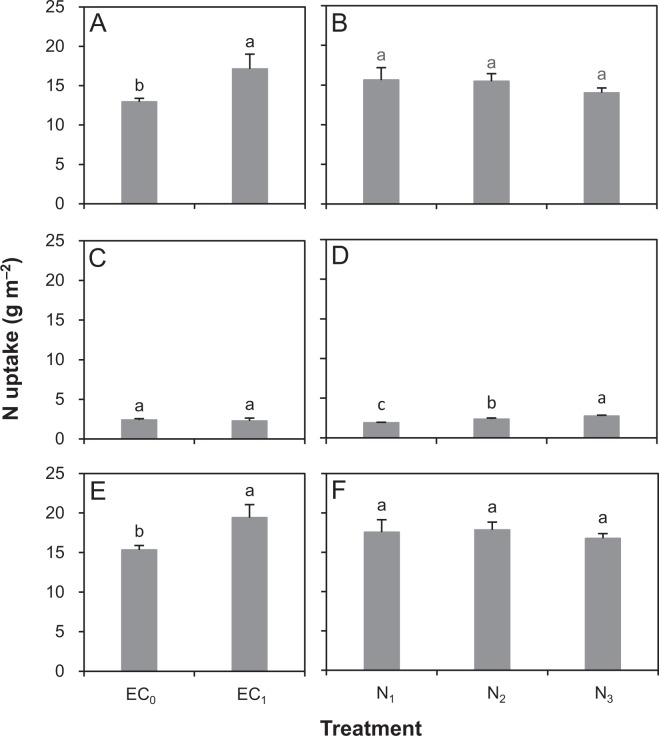
Uptake of non-fertilizer N (**A**,**B**), fertilizer N (**C**,**D**), and total N (**E**,**F**) in aboveground biomass in a rice cultivar Liangyoupeijiu as affected by earthworm castings (EC_0_: 0 g m^−2^; EC_1_: 17 g m^−2^) and N rates (N_1_: 9 g m^−2^; N_2_: 12 g m^−2^; N_3_: 15 g m^−2^) in 2016. The interactive effect between earthworm casting treatment and N rate on these parameters were not significant. Error bars represent SE (*n = *9 for each earthworm casting treatment, and 6 for each N rate). Within each graph, columns with the same letters are not significantly different at the 0.05 probability level (LSD test).

Non-fertilizer N uptake was 32% higher under EC_1_ than EC_0_ (Fig. 2A). There was no significant difference in non-fertilizer N uptake among N_1_, N_2_ and N_3_ (Fig. 2B). Fertilizer N uptake was not significantly different between EC_1_ and EC_0_ (Fig. 2C), while it was slightly but significantly increased with an increasing rate of N (Fig. 2D). EC_1_ had a 27% higher total uptake of N than did EC_0_ (Fig. 2E). The difference in total N uptake was, however, insignificant among the three N rates (Fig. 2F).

## Discussion

Our results showed that application of earthworm castings produced during the oilseed rape-growing season in the rice-oilseed rape fields had a significant positive yield effect on rice. This finding is in agreement with our observation in a pot experiment^12^. These works support the previously-expressed viewpoints that oilseed rape is an excellent previous crop for cereals^8–10^ and that earthworm presence in agroecosystems can lead to increase in crop yield^24^.

Analysis of yield components indicated that the yield increase effect of the application of earthworm castings was mainly attributed to an enhancement of sink size that resulted from increased panicle number. This finding is not in agreement with the predominant standpoint among rice breeders that increasing panicle size is the most promising approach to enhancing sink size and consequently improving rice yield potential^16^.

More interestingly, the increased panicle number induced by the application of earthworm castings did not cause a significant decrease in panicle size in this study. This result is inconsistent with previous studies, such as Ying *et al*.^14^ and Huang *et al*.^15^, which reported that there was a compensation between the two yield components. Moreover, in the present study, the enhanced sink size was achieved not at the expense of spikelet filling percentage and grain weight in rice that has been grown with earthworm castings applied. These results demonstrated that compatible relationships among yield components were established in rice with the application of earthworm castings. In this regard, it is suggested that increasing biomass production is a feasible way to decouple the compensations among yield components in cereals including rice^25,26^. In this study, a higher total aboveground biomass was achieved in rice applied with earthworm castings, which could be responsible for the compatible relationships among yield components in rice with the application of earthworm castings.

On the other hand, the results of total aboveground biomass and harvest index revealed that there was a positive yield effect from the application of earthworm castings which was mainly driven by increased total aboveground biomass rather than harvest index. This is not surprising because it has been well-documented that there is little scope to further increase in harvest index under favorable conditions^18–20^. However, perhaps interestingly, the application of earthworm significantly did not decrease and even slightly increased harvest index. This is different from the effect of application of chemical fertilizer N on rice, in which harvest index is generally decreased^27^. Harvest index is determined by the remobilization of stored reserves into the growing grain and the transient photosynthesis during grain formation^28^. An increase in the former is usually achieved accompanied with early senescence and shortened grain-filling duration, which can be induced by unfavorable conditions such as water stress^29^. However, this must not be the case in the present study, because we observed that the application of earthworm castings made the rice leaves greener during the grain-filling period. Therefore, the slightly increased harvest index might be related to the transient photosynthesis during grain formation in rice applied with earthworm castings in this study. This highlights that further investigations are needed to determine the effect of application of earthworm castings on photosynthetic characteristics during the ripening period in rice.

Decreasing N rate from 150 to 90 kg ha^−1^ did not result in significant yield reduction in the hybrid rice cultivar Liangyoupeijiu in 2016. Huang *et al*.^30^ determined N response of this cultivar over a wide range of N rates (60–410 kg ha^−1^). Their results showed that Liangyoupeijiu required a minimum total N rate of 120–150 kg ha^−1^ to produce maximum grain yield. These suggest that the hybrid rice Liangyoupeijiu does not necessarily need more N fertilizer to produce high grain yield. Consistent with this, Huang *et al*.^31^ have observed that higher grain yield in hybrid rice is mainly driven from a higher grain yield without N fertilizer rather than increases in grain yield with N fertilizer. This suggests that greater application of N fertilizer is not needed to benefit from hybrid production and that improving and maintaining soil fertility should be the focus for sustaining hybrid rice production. This can be further supported by this study’s results in 2016 that the total N uptake mainly depended on non-fertilizer N uptake in the hybrid rice cultivar Liangyoupeijiu. The non-fertilizer and fertilizer N uptake accounted for 87% and 13% of the total N uptake, respectively (Fig. 2). In addition, the positive effect of application of earthworm castings on panicle number, total aboveground biomass, and grain yield in 2016 also could be explained by an increase in non-fertilizer N uptake. The increased non-fertilizer N uptake under application of earthworm castings was partially due to that the earthworm castings contained a certain amount of available N.

Significant cultivar differences in grain yield were detected in 2017. Inbred cultivars generally produced lower grain yields than did hybrid cultivars. The lower grain yields of inbred cultivars were mainly attributed to lower grain weight and to lower total aboveground biomass. When comparison was made among the hybrid cultivars, the lowest grain yield was recorded in Liangyoupeijiu, which was released in 1999 (Table 1). The yield difference was small among the other six hybrid cultivars, which were released during 2008 to 2016 (Table 1). It seems that the breeding effort did not contribute much to increased rice yield in the past decade. Also interestingly, the six high-yielding hybrid cultivars could be divided into four groups according to their yield component performance: (1) Longliangyou 97, Xiangliangyou 396 and Y-liangyou 1 are characterized by more panicle number, (2) Y-liangyou 2 is distinguished for its large panicle size, (3) Shenliangyou 5814 has a higher spikelet filling percentage, and (4) Zhunliangyou 608 is notable for its large grain size. This suggests that there are multiple strategies or pathways for developing high-yielding hybrid rice cultivars.

## Conclusions

Application of earthworm castings produced during the oilseed rape-growing season in rice-oilseed rape cropping fields had a significant positive yield effect on rice. This was attributed to increased panicle m^−2^ and total aboveground biomass while spikelets panicle^−1^, spikelet filling percentage, grain weight, and harvest index were not affected.

## Methods

Field microplot experiments were conducted in a rice field at the research farm of Hunan Agricultural University (28°11′N, 113°04′E) in Changsha, Hunan Province, China in 2016 and 2017. The soil of the rice field was a tidal clay (Fluvisol, FAO taxonomy) with the following properties: pH = 5.75, organic matter = 34.2 g kg^−1^, available N = 81.6 mg kg^−1^, available P = 34.4 mg kg^−1^, and available K = 56.7 mg kg^−1^. The soil test was based on samples taken from the 0–20 cm layer before the experiment was begun in 2016.

In 2016, eighteen microplots were constructed by inserting bottomless PVC boxes (40 cm long × 40 cm wide × 30 cm deep) into the soil to a depth of 20 cm with a collar of 10 cm aboveground. A hybrid rice cultivar Liangyoupeijiu was grown a factorial combination of absence (EC_0_: 0 kg m^−2^) and presence of earthworm castings (EC_1_: 17 kg m^−2^) with three N application rates (N_1_: 9 g m^−2^; N_2_: 12 g m^−2^; and N_3_: 15 g m^−2^). The earthworm casting amount of EC_1_ was based on an estimate obtained by multiplying daily production rate of earthworm castings (78 g m^−2^ d^−1^) by the duration of an oilseed rape-growing season (218 d). The daily production rate of earthworm castings was obtained on the first day after harvesting the oilseed rape from 10 randomly selected 1-m^2^ plots in a rice-oilseed rape cropping field located in Nanxian (29°21′N, 112°25′E), Hunan Province, China in 2015. The N rates were chosen according to the local recommended N rate (150 kg ha^−1^) for rice production in the study region. The treatments were arranged in a completely randomized block design with three replications.

In 2017, seventy-two microplots were constructed using the same procedures as described above. Nine rice cultivars, including two inbred cultivars (Guihefeng and Huanghuazhan) and seven hybrid cultivars (Liangyoupeijiu, Longliangyou 97, Shenliangyou 5814, Xiangliangyou 396, Y-liangyou 1, Y-liangyou 2 and Zhunliangyou 608), were grown under EC_0_ and EC_1_ with N_2_. The N rate was chosen according to the results in 2016, when grain yield was not significantly different among the three N rates (Table 1). The treatments were laid out in a split-plot design with earthworm casting treatments as the main plots and cultivars as subplots. The experiment was replicated four times. All the cultivars used in this study are ones that have been widely grown by rice farmers in southern China.

The earthworm castings used in the experiment were collected from rice-oilseed rape cropping fields located in Nanxian after harvesting the oilseed rape in 2016 (Fig. 1C). The site has a moist subtropical monsoon climate with an annual average temperature of 16.6 °C, an annual average rainfall of 1238 mm, and an annual average sunshine duration of 1776 h. The soil in the fields is a purple calcareous clay (Fluvisol, FAO taxonomy). The dominant earthworm species in the field is *Pheretima guillelmi*. The earthworm castings had the following properties: pH = 7.89, organic matter = 61.4 g kg^−1^, available N = 128 mg kg^−1^, available P = 44.2 mg kg^−1^, and available K = 254 mg kg^−1^. The N fertilizer used in 2016 was ^15^N-labeled urea (5.18% isotopic abundance, provided by Shanghai Institute of Chemical Industry, China), and unlabeled urea in 2017.

Pre-geminated seeds were sown on a seedbed on 10 May. Seedlings were transplanted on 5 June. Transplanting was done with four hills per microplot and one seedling per hill. Earthworm castings were applied at 1 day before transplanting. N fertilizer was split-applied with 50% as basal (1 day before transplanting), 30% at early tillering (7 days after transplanting), and 20% at panicle initiation. Superphosphate (4.8 g P_2_O_5_ m^−2^) was applied as basal fertilizer. Potassium chloride (8.4 g K_2_O m^−2^) was split equally at basal and panicle initiation. A floodwater depth of about 5 cm was maintained in the microplots until 7 days before maturity, when the microplots were drained. Insects, disease, and weeds were controlled by using approved pesticides to avoid yield loss.

Plants were sampled for each microplot at maturity in both years. Panicle number was counted in each hill to determine panicles m^−2^. Plants were separated into straw (including rachis) and spikelets by hand threshing. Filled spikelets were separated from unfilled spikelets by submerging them in tap water. Dry weights of straw and filled and unfilled spikelets were determined after over-drying at 70 °C to constant weight. Three subsamples of 30 g of spikelets and all unfilled spikelets were taken to count the number of spikelets. Total aboveground biomass was the total dry matter of straw and of filled and unfilled spikelets. Spikelets panicle^−1^, spikelets m^−2^ (panicles m^−2^ × spikelets panicle^−1^), spikelet filling percentage (100 × filled spikelet number/total spikelet number), grain weight, and harvest index (100 × filled spikelet weight/total aboveground biomass) were calculated. Grain yield was adjusted to a moisture content of 0.14 g H_2_O g^−1^.

In 2016, the dried plant samples were ground into fine powder for determining their N content (VAP50 Kjeldahl meter, Gerhardt, Königswinter, Germany) and ^15^N abundance (Delta V Advantage isotope mass spectrometer, Thermo Fisher, Waltham, MA, USA). Total N uptake, and uptake of fertilizer and non-fertilizer N in aboveground biomass were calculated according to Huang *et al*.^32^.

Data were analyzed by analysis of variance with the use of Statistix 8.0 software (Tallahassee, FL, USA). In 2016, the statistical model included replication, earthworm casting treatment, N rate, and the interaction between earthworm casting treatment and N rate. In 2017, the statistical model included replication, earthworm casting treatment, cultivar, and the interaction between earthworm casting treatment and cultivar. Means were compared based on the least significant difference test (LSD). The 0.05 probability level was used to test for statistical significance.

### Data availability

All data generated or analysed during this study are included in the article.
